# An Intervention-Focused Model for Understanding Fulfillment Among Older Adults

**DOI:** 10.1093/geroni/igaf122.3703

**Published:** 2025-12-31

**Authors:** Joy Collins, Carly Woods, Lance Kruse, Benton Smith, Jeremy Cochran, Lisa D’Ambrosio

**Affiliations:** Humana, Louisville, Kentucky, United States; Humana, Louisville, Kentucky, United States; Humana, Louisville, Kentucky, United States; Burke, Inc., Cincinnati, Ohio, United States; Burke, Inc., Cincinnati, Ohio, United States; Massachusetts Institute of Technology, Cambridge, Massachusetts, United States

## Abstract

Older adults experience a range of health issues as they age. Many community and policy initiatives focus primarily on physical health, overlooking the concept of fulfillment — the satisfaction of a life well-lived and needs being met. Prior studies have shown that fulfillment and purpose in life are linked to reduced anxiety and depression, which in turn support better physical health and lower chronic disease risk. Our study aimed to develop and validate a model of fulfillment among American adults aged 62+, using both qualitative (expert interviews, discussions, n = 23) and quantitative methods (survey, n = 1150). The qualitative phase clarified what fulfillment means to older adults, while quantitative analysis using Bayesian Network Analysis identified the key factors and drivers of fulfillment. In the second research phase (n = 5500; completion August, 2025), we will validate the model in a larger sample and analyze fulfillment among key subgroups. Average fit was tested by introducing data noise across 100 iterations. The model demonstrated good reliability (average fit score = 72.1%, SD = 5.8%) and all items within each factor achieved statistical significance (p < .01). A Bayesian Network structural model predicted fulfillment agreement ratings with factors as the independent variables: Precision = 70.7%, ROC Index = 89.8%. By identifying the activities and emotions that drive fulfillment, this research offers tools for policymakers and healthcare providers to design more effective, patient-centered interventions. Ultimately, measuring and improving fulfillment can enhance the well-being and health of older adults, guiding targeted support where it is most needed.

